# Southern Surgical and Gynecological Association

**Published:** 1893-12

**Authors:** 


### *SOCIETY REPORTS.*

---

### SOUTHERN SURGICAL AND GYNECOLOGICAL ASSOCIATION.

---

ABSTRACT OF THE PROCEEDINGS OF THE SIXTH ANNUAL MEETING, HELD IN NEW ORLEANS, LOUISIANA, NOVEMBER 14, 15 AND 16, 1893.

---

### FIRST DAY—MORNING SESSION.

The Association convened in the assembly room of the Medical Department of Tulane University, and was called to order by the President, DR. BEDFORD BROWN, of Alexandria, Virginia, at 10 A. M.

The reading of papers was proceeded with, and the first paper read was by DR. HOWARD A. KELLY, of Baltimore, entitled

### DIAGNOSIS OF PELVIC INFLAMMATORY DISEASES.

He called attention to certain common sources of error in making diagnosis of pelvic inflammation. An erroneous conclusion is often reached in these cases both by general practitioner and specialist by relying for the diagnosis upon such symptoms as dysmenorrhea, more or less persistent pain in the pelvis, attacks of pain confining the patient to bed, diagnosed as peritonitis, difficult locomotion, cachexia (due to morphine habit), tenderness on pressure over the ovarian region, and extreme tenderness at the vault in a vaginal examination.

In order to make a diagnosis of true pelvic inflammatory disease, the inflamed structures must be examined directly by touch. The various subjective symptoms must be regarded as of secondary importance in reaching a diagnosis.

Even the patient's observation that she has passed a quantity of pus cannot be relied upon unless the pus is seen by the physician, as patients often mistake muco-purulent discharges from the uterus for the emptying of an abscess.

The direct examination, the sole test, is made by the vagina, or by the vagina and lower abdominal wall, and the diagnosis of pelvic inflammatory disease is made when a definite hard resisting mass is felt on one or both sides of the cervix.

Through an empty rectum these masses are still more distinctly outlined. When the disease is not quite so evident, a bimanual examination through the rectum and abdomen should be made, carrying the index finger of the lower hand above the rectal pouch behind the uterus and laterally out on to the broad ligaments. The most minutely accurate examination of the pelvic organs which can possibly be made is called for when the ovaries and tubes are enclosed in delicate bands of adhesions which allow considerable mobility to structures not enlarged. This is accomplished by the trimanual method by vagina, rectum and abdomen simultaneously, under anesthesia.

DR. C. KOLLOCK, of Cheraw, S. C., followed with a paper entitled

THE CONSERVATIVE TREATMENT OF PYOSALPINX.

He said in cases of pyosalpinx, much caution and a very careful and rigid examination are called for to determine the cause of the presence of pus, the length of time it has been there and the condition of the walls of the tube in which it is found. Attention should also be given to the peritoneum and ovaries, but above all, there should be the strictest inspection of the endometrium, a disordered condition of which contributes much to the production and continuance of pus in the tubes.

Within a year or two changes have been made in the treatment of pyosalpinx, and conservatism now enters largely into its management. Men of high position in the profession are more decidedly agreed that a moral obligation rests upon us to relieve patients without the sacrifice of any organ, or part of one, when this is compatible with safety. Recently Polk, Pryor, Krug, Boldt and Dudley had reported to the New York Obstetrical Society a number of cases of pyosalpinx treated by the conservative method now in vogue. This treatment, when faithfully carried out by curettement and aseptic divulsion, has not only been successful in saving the tube and ovary on the non-affected side, but in several instances the diseased tube was entirely relieved of the presence of pus. That many cases of pyosalpinx have been accurately diagnosticated and radically cured without the mutilation of any part of the sexual organs, is well authenticated. Dr. Kollock's experience, while limited compared to that of others, has been sufficient to convince him that the conservative system of practice is bringing us to that period when the mutilation of women, once supposed to be necessary, should cease.

DR. JOSEPH PRICE, of Philadelphia, alluded to dropsical tubes as being a group of cases that puzzled the practitioner from a diagnostic point of view, and later surgically. Angry pus cases, while acute in their early history, were simply cases to be dealt with surgically. The attacks of pain were numerous, and fixation and tenderness characteristic symptoms. Everything in the pelvis was board-like, and when the surgeon got into the abdomen from above it was difficult to distinguish the uterus from the appendages, and *vice versa*. These were trying cases to deal with.

DR. JOHN D. S. DAVIS, of Birmingham, Ala., said he was in favor of evacuating pus wherever it was found in the body. There were, however, some cases in which pus could be removed without sacrificing the ovaries or tubes. As to the use of an anesthetic, he considers it absolutely essential in the examination of doubtful cases, but where the diagnosis is plain it is not necessary.

DR. R. B. MAURY, of Memphis, said the great difficulty in the class of cases referred to by Dr. Kollock, in which there was pelvic inflammation associated with muco-purulent discharges from the uterine cavity, was to decide whether there is a pyosalpinx. We have what is denominated endometritis, associated with it normal discharges and exudation in the pelvis. But Dr. Maury says he is at a loss sometimes with the most careful diagnostic measures, whether under ether or without it, to form in his own mind an accurate picture of what the precise state of things is in the pelvis. The rule he has laid down in the treatment of such cases is, if they are acute, non-puerperal or puerperal, that the woman is entitled to a certain period of rest and other measures non-surgical, before deciding upon a radical operation.

DR. L. S. MCMURTRY, of Louisville, Kentucky, deliverd a memorial address on Dr. Ephraim McDowell.

### FIRST DAY—AFTERNOON SESSION.

### THE INCISION IN ABDOMINAL SECTION—HOW TO CLOSE IT—POST-OPERATIVE COMPLICATIONS ABOUT IT.

DR. JOSEPH PRICE, of Philadelphia, read a paper on this subject. He said the question that most vitally concerns surgical and gynecological work is, how can the mortality be reduced? Surgical judgment and surgical fingers repeatedly determine the issue of life or death.

*The Incision.*—We have nothing from which we can ever approximately determine to what extent the length of the incision influences the mortality. The statistics of comparative results would not prove satisfactory, for the reason of the entry of so many other compromising elements, adhesions, their character, extent and locality. That the incision exercises a greater influence than is generally recognized or admitted, he entertained little doubt. As to length, no rule of mathematical certainty could be laid down. In his own experience he finds the balance of both convenience and safety to lie with the short incision. The short incision narrows the limits of hemorrhage. It is safe to begin with a small incision, and where the size and character of the tumor or complications present require a larger one, it can easily be made. Very much abdominal work can be done through an opening admitting only two fingers. The reliance of the abdominal surgeon must be largely in educated fingers. In the majority of cases an operation can be done through a small incision without the operator or spectators seeing viscera. Universally adherent irreducible or solid tumors require a long incision for delivery and for dealing with complications that can only be dealt with through a long incision, those beneath and on the sides of tumors. In the majority of cases to so enlarge the opening as to obtain a view of the parts, we augment the risk of ventral hernia and provoke tedious convalescence.

The importance of a perfect closure of the incision has only recently received that attention it deserves. The effort should be to approximate as nearly as possible normal conditions, anticipating and dealing with all existing or possible complications with scrupulous minuteness and care, thus guarding against those incidents which are too frequent. He would not pretend to suggest uniform procedures to be carried out in all cases, as each operator has his own way and does his own work best that way, and it would not be possible for him to apply the methods of others safely and successfully without special training.

He is satisfied that the exposure and manipulation of the incision as well as the peritoneum is harmful. Incisions bathed in pus, and filth and freely manipulated often refuse to unite. Suppurating wounds are largely due to careless closure or to tight sutures, including too much tissue. Tight suturing is too common, and has destroyed life in many feeble subjects. Suppuration due to tight suturing and stitch-hole abscesses, in all sections, where they do not result fatally, prolong convalescence. Cases were cited in point.

Through and through suturing, including all structures, more of the central structure than skin or peritoneum, with either silkworm gut or pure silk, give and continue to give the most satisfactory results. Silkworm gut seems to be the favorite material at present, as it possesses all the natural and essential qualities of a suture, is small, strong and non-irritating, the three cardinal virtues of all good suturing material. Terracing sutures has nothing to recommend it; on the other hand, Dr. Price believes it prolongs an operation. Retraction of skin and peritoneum by the introduction of silkworm sutures gives inclusion to more central structures and the least possible tension on skin and peritoneum. Keith, Tait and Bantock all use a fine straight needle, and their work has been about perfect. The use of large, curved cutting needles is harmful, their use primarily favors hemorrhage and secondary stitch-hole abscesses.

DR. L. S. MCMURTRY, of Louisville, demonstrated his method of suturing on the board. He brings peritoneum to peritoneum, muscular structure to muscular structure, fascia to fascia, skin to skin, and says the least quantity of interposing material that we have between the tissues that are to be brought together the better. He disagrees with Dr. Kelly that the drainage tube is the cause of hernia after closing the incision.

DR. R. B. MAURY, of Memphis, favored the silkworm gut suture. His experience covers nearly three hundred sections, and he has simply used the through and through suture. He has had almost no abscesses and the fewest possible number of hernia, which, he says, can be counted on the fingers of one hand.

DR. T. J. CROFFORD, of Memphis, said it was considered that all hernias resulting from abdominal section were due to failure to get union between the opposing layers of transversalis fascia. He uses a long, curved needle instead of a straight one. With it he can put in stitches in one-third of the time he can with the ordinary needle. He has used it in upwards of 200 sections, and has not had a case of hernia to follow one of them. He has also had the fewest number of stitch abscesses.

### IS OPERATION DEMANDED IN ALL CASES OF APPENDICITIS?—THE BEST TIME TO OPERATE.

This paper was read by DR. A. M. CARTLEDGE, of Louisville, Ky. He said that inflammatory conditions of the appendix are essentially intra-peritoneal lesions. Modern surgeons have an abiding faith in the surgical maxim that whenever pus is believed to be present in tissues or organs of the body, it should be removed; hence the new pathology of a very old and frequently fatal malady inspired surgeons to attempt some radical means of relief. Perfection in technique can only come from individual experience and a knowledge of the work of others.

The pathology of a disease is the only true keynote to its rational treatment. Probably the best classification of appendicitis is catarrhal (simple); ulcerative (from tuberculosis from foreign bodies); perforating (from ulcerative perforation, from strangulation, the result of twisting). This classification deals strictly with the changes occurring in the appendix, and should be considered apart from the peritoneal and other conditions which may ensue and cause well-marked variations in the clinical course of the disease. If the walls of the appendix give way in a mass of fibrous adhesions, the result of long continued irritation, the pus which forms is rather securely encapsulated, and may be days, weeks, even years, finding an outlet. In fact, as is often the case, if the bacillus coli communis predominates and a few staphylococci are present, it may remain incapsulated unless it receives a new impetus of irritation. Cases were reported illustrating this point. Cases were also reported illustrating the part played by injury as an exciting cause in appendicitis, and the belief was expressed that a chronic form of unrecognized appendicitis existed prior to such injury.

We know more about the pathology of ulcerative or suppurative appendicitis than we do of the catarrhal form, because the cases not operated upon which recover are mostly called catarrhal. These are cases which progress with little pain, with very little fever, 101° F. as a maximum, and have a tumor which subsides. These cases are the pride of the poultice and the opium practitioner. Ulcerative appendicitis must be either tuberculous or traumatic, the trauma consisting of foreign bodies and enteroliths, usually the latter. The tuberculous would only give rise to acute symptoms as the result of cicatrization and stenosis, with distal distension or secondary inflammation with pus organisms. Either of these results favor perforation. This is essentially the chronic variety, but will eventually lead to perforation, probably in the ways indicated.

When physicians come to view inflammations of the vermiform appendix in their proper light, the author said the prognosis will assume a very different shade. We should consider any appendix once so affected as to deserve the name of appendicitis, whether from tubercle or trauma, a lastingly diseased structure, and the fancied cures are quiescent states, the results of very easily recognized conditions. If we could trace our so-called first attack cases of appendicitis through subsequent ones, we would say the progress not only as to health and comfort, but as to, life is bad, very bad. A man has the trouble three, four or five times, apparently recovers, all counted as cures probably by different physicians. Finally he dies in an attack; the death is counted but once and sometimes not then, for if, as is often the case, death results from the rupture of an unrecognized appendicitic abscess, or from diffuse peritonitis after perforation, the chances are that the cause is never suspected and death is recorded as occurring from peritonitis. Every case of appendicitis not barred by surgical limitation should be operated upon. The best time, provided the symptoms are not too urgent, is after the bowels have been thoroughly moved.

Dr. Joseph Price agreed with the author of the paper that there was but one treatment for appendicitis, namely, removal of the appendix. He considers it a murderous disease, to be classed with extra-uterine pregnancy. Both demanded prompt surgical treatment when first discovered. He recommends in acute cases of appendicitis without pus removal of the appendix and freeing of the inflammatory adhesions.

Dr. G. W. Long, of Richmond, opposed operation in every case of appendicitis. Autopsies had shown that one-third of the human race had had at some period of their lives this disease. That being true, and considering the small per cent. of deaths, it naturally follows that appendicitis does not always kill, even if it is not operated on. In the catarrhal form he thinks that there is no reason for operating. In the perforative form we should operate. In the perforative form without adhesions we should also operate as soon as we make a diagnosis.

Dr. William T. Briggs, of Nashville, had been operating on every case of appendicitis that came into his hands where the diagnosis was clearly established, and he has had no occasion to regret it. He has operated in cases where there were perforative symptoms, and in others where there were none; in some where there were, and in others where there were not, suppuration, and still others where there was, and where there was not, sloughing.

DR. HUNTER McGUIRE, of Richmond, said he had many a time operated too late, but never in his life had he operated too soon. If, after free and full purgation with salts, administered by the mouth and rectum, the symptoms are not relieved, he thinks the time for operation has come and does not hesitate to operate. He has never known the mere operation in the hands of skillful surgeons to kill or add to the danger of the patient's life. Appendicitis kills, and it is put down to inflammation of the bowels, peritonitis or something else.

TREPHINING AS A CURE FOR TRAUMATIC EPILEPSY.

By DR. JOHN T. CHAPMAN, of Bessemer, Ala.: He operated on a boy, who, some years previously, had received an injury of the head by a blow fracturing the parietals. Three weeks after the injury he began to have epileptic convulsions. At the time Dr. Chapman saw the patient the convulsions had become more frequent, and with greater force. Two buttons of bone were removed at the seat of injury; there was considerable pressure from depressed bone; the membranes were hard and indurated. The indurations were cut out and edges of dura brought together by continuous suture. The wound healed by first intention. For two months after the operation the patient continued to have convulsive seizures, but they gradually grew less until they ceased. The operation was done four and a half years since. The patient is eighteen years old, weighs 170 pounds, and works in a foundry. He believes the doctrine that depressed fractures of the skull without symptoms require no operative interference is in a measure responsible for many of the unfortunate sequelæ of head injuries.

DR. B. E. HADRA, of Galveston, Texas, read a paper entitled

SOME REMARKS ON THE SURGICAL TREATMENT OF EPILEPSY.

He thinks that modern researches promise to divest even the so-called genuine epilepsy of its mysterious functional character, and to make it consequently more accessible to surgical interference. Among the points he would mention as having to be cleared up is the deficiency in the knowledge of the great number of brain centers that must exist. As an instance he mentions the unquestionable fact that very often the stomach or the intestines give the initial symptoms, but because we do not yet know these centers, and because signals are very abstruse, it may easily happen that another group of muscles, which only secondarily become excited, is charged with giving the signal. The next point is to find the real seat of the primary morbid changes in the brain, which is not necessarily the focus belonging to the initial signal. Topographical and electrical localization map out only the latter. He insists that the induced current used in a different way will be all we may desire for such a purpose. It must be applied over a large area of the exposed cortex until a spot is met from where a certain group of muscles not only can be made to contract, but from where a regular epileptic fit can be elicited. This spot must be the locality of the morbid substratum, whether it coincides with the physiological focus of the muscles giving the signal or not; consequently this spot must be removed.

He concludes his paper with the proposition to have uniform blanks for operations on the brain, and to have the questions filled by a critical friend during the operation in order to avoid neglect and to prevent post-operative imagination from playing its obliging part in the adjustment of the historical data.

DR. WILLIAM T. BRIGGS, of Nashville, followed with a paper entitled

### PERSONAL EXPERIENCE IN THE OPERATIVE TREATMENT OF STONE IN THE BLADDER.

He said living in the midst of the stone region, and in a city whose celebrity as a surgical center has been long established, it has been his fortune to have met with an unusually large number of cases of vesical calculi. He had had two hundred and eighty-four cases of stone under observation during the past forty-two years. The Southern States had furnished the greater number of cases; a few had come from Western States. Tennessee, Kentucky and Alabama have supplied the largest number; but Georgia, Florida, Texas, Arkansas, North Carolina, Virginia, Missouri and Illinois have contributed cases. Two hundred and seventy-two of the nnmber were males, twelve females. One hundred and fifty-three were children, or youths under twenty years of age ; one hundred and thirty-one were adults, varying in age from twenty-one to eighty.

In operations for stone he had not restricted himself to any single method. He had done all of the operations, both cutting and crushing, and he considers it very fortunate that surgery has so many resources for the relief of this distressing and painful malady. The success of every method of operating largely depends on the preparatory treatment of the patient. The pre-eminent success of Dudley, Mott and others was doubtless due to the judicious treatment employed in the preparation of subjects for the operation, and Dr. Briggs is sure that his own success has been greatly enhanced by a strict observance of the preparatory treatment.

In conclusion, Dr. Briggs said his experience in the surgical treatment of stone in the bladder would sustain the following propositions :

1. No method of operation is adapted to all cases.
2. Thorough preparatory treatment is essential to success.
3. Litholapaxy is *the* operation when the patient is an adult with a capacious and tolerent urethra, with a bladder free from severe chronic cystitis, and with a small or medium sized stone or, if large, of soft consistence.
4. The suprapubic is the best operation for large and hard calculi.
5. The medico-bilateral should be chosen in all other conditions because it is the easiest, safest and best.

DR. WILLIS F. WESTMORELAND, of Atlanta, read a paper on the

### TREATMENT OF GUNSHOT WOUNDS.

The first of this class of injuries that he was called upon to treat was shortly after he graduated, and it thoroughly convinced him of the fallacy of probing. He never uses a probe in a gunshot wound except as it may become necessary in the progress of a formal antiseptic operation. The probing rarely ever does any good beyond satisfying a morbid curiosity. Even if the wound is not infected by the probe itself, it allows the entrance of air. It destroys nature's occlusive blood clot and in this way prevents prompt union.

WYETH'S BLOODLESS METHOD IN AMPUTATION AT THE HIP.

By DR. F. W. PARHAM, of New Orleans. Dr. Parham prefaced his own paper by reading some extracts from a paper by Dr. John A. Wyeth, read before the last meeting of the New York State Medical Association. He made use also of the statistics kindly furnished him by Dr. Wyeth. These statistics were as follows: Sarcoma, 17 cases, 2 deaths; 11.76 per cent. Inflammatory bone disease, 18 cases, 3 deaths; 16.6 per cent. Violence, 4 cases, 4 deaths; 100 per cent Nerve injury, 1 case, 0 death. Total for disease, 36 cases, 5 deaths; 13.88 per cent. For injury, 4 cases, 4 deaths; 100 per cent. For both disease and injury, 40 cases, 9 deaths, or a total of 22.5 per cent. In this list is one case now published for the first time. Boy, aged three, sarcoma of thigh, recurrent, amputated by Wyeth's method by Dr. Parham October 5, 1893, discharged cured October 24, 1893. These statistics show a mortality reduction for civil practice of at least one-half. Ashhurst's statistics gave 40.2 per cent. for disease, 82.4 per cent. for injury, or a total of 64.1 per cent. Leming gives gunshot wounds 98 per cent., disease 42 per cent.

Dr. Parham referred to the various methods proposed for controlling hemorrhage at the hip and spoke of the various modifications of the Wyeth method. He specially urged that the outer pin should be placed higher, so that the disarticulation might be done before the tube was removed. He favored the suggestion of Thomas that in placing the pins, the tube should be put around first at the proper place, and that then the pins should be put in at the lower border of the tube. He believed that the bone should be disarticulated entirely without sawing. In conclusion, the reader remarked: "I am inclined to agree with Murdoch that the method of Wyeth is the best yet devised."

DR. J. MCFADDEN GASTON, of Atlanta, Ga., read a paper entitled

OPERATIVE PROCEDURES FOR CARCINOMATOUS TUMORS OF THE BREAST.

See page 579.

DR. JOHN T. WILSON, of Sherman, Texas, read a paper entitled

DOES GONORRHEA IN THE FEMALE INVARIABLY PREVENT CONCEPTION?

He said it has long been known that gonorrhea in the female was sometimes attended with complications that proved troublesome and of serious import. Authors had for many years been describing endometritis, metritis, inflammations of the tubes, ovaries and peritoneum produced by an ascending specific vaginitis, these structures being invaded by the poison, it slowly creeping up through the cervix, involving first the mucous membranes in its track and extending by continuity of structure to the deeper tissues. The more serious results, however, were not appreciated nor so well understood until within recent years, when laparotomy became so common an operation, and the pathology of the more important sequelæ were studied from the specimens themselves. According to the experience of our best authorities it is so difficult to positively differentiate between gonorrheal and severe simple vaginitis without a clear and authentic history, it being attended with the same symptoms and the properties of also infecting the male, that it is not altogether an easy task to say when ovarian, tubal and uterine troubles, even with the presence of the Neisser gonococcus, have a specific origin, especially as simple vaginitis will sometimes produce them all. Dr. Wilson had observed quite a number of women who were the victims of gonorrheal infection, many of them innocently so, having contracted it from their husbands, and believed it to be an ordinary leucorrhea; many of those whose history he was enabled to follow afterwards bore children for many years, were apparently healthy and gave no evidence of the usual complications.

Dr. Wilson then reported cases illustrative of some of these conditions and results. That gonorrhea does frequently prevent conception is probably well established; but he does not think it is by any means the universal rule, clinical illustrations are too many to the contrary. If Noeggerath's statements are literally true, sterile women and fruitless marriages would be far more common and the increase in the race would be greatly lessened, for there are a surprisingly large percentage of men, judging from his experience, who, if they confessed the truth, have suffered at some time in their lives with gonorrhea.

### SOME REMARKS ON THE PRACTICAL TREATMENT OF HEPATIC ABSCESS.

By DR. JAMES E. THOMPSON, of Galveston, Texas. The author confined himself to a few practical remarks on the diagnosis and treatment of hepatic abscess, and reported two interesting cases. He mentioned a few points on the treatment of the cavity after the contents had been successfully removed. It is often exceedingly difficult to obtain free drainage, even when large tubes are employed. In some cases, even swabbing the walls of the cavity is ineffectual, and these cases are practically hopeless, owing perhaps to inherent tissue weakness. The author tried curetting in one of his cases, and although he removed, as he thought, all necrotic tissue, still in a few days the cavity was as bad as before. Continuous irrigation often affords a means of efficient removal, but the tubes have an aggravating habit of becoming blocked, necessitating frequent changing and cleaning. Irrigation with a solution of sulphate of quinine—1-1000—was in one of his cases remarkably successful, and although the improvement may have been a coincidence and not a *post hoc ergo propter hoc*, he thinks that in cases of amœbic dysentery, at least, it deserves a fair trial.

